# 改良POSSUM评分在预测老年非小细胞肺癌手术风险中的应用价值

**DOI:** 10.3779/j.issn.1009-3419.2014.09.05

**Published:** 2014-09-20

**Authors:** 蓉 王, 德伟 高, 卫琴 龚, 致如 梁

**Affiliations:** 100853 北京，解放军总医院南楼综合外科 Surgery Department of Nan-lou, Chinese PLA General Hospital, Beijing 100853, China

**Keywords:** 肺肿瘤, 老年人, 手术风险, POSSUM评分, Lung neoplasms, Elderly, Operation risk, POSSUM scoring system

## Abstract

**背景与目的:**

对于评价老年患者能否耐受肺癌手术，目前尚无明确标准。本研究旨在探讨改良POSSUM（Physiological and Operative Severity Score for the Umeration of Mortality and Morbidity）评分在预测老年非小细胞肺癌（non-small cell lung cancer, NSCLC）患者术后并发症发生率和病死率中的应用价值，为手术治疗的决策提供重要依据。

**方法:**

2007年12月-2013年12月在解放军总医院接受手术治疗的老年NSCLC患者138例，其中男性88例，女性50例，收集临床资料，各因素对术后实际并发症发生率和病死率的影响，采用二值多元*Logistic*回归分析。在有、无并发症两组中，采用成组t检验对标准及改良POSSUM评分值进行比较。绘制标准POSSUM和改良POSSUM的受试者工作特征曲线（receiver operating characteristic curve, ROC），计算曲线下面积（area under the curve, AUC），两组间AUC比较采用*t*检验。计算改良POSSUM评分预测值和实际并发症发生率和病死率的符合度。

**结果:**

共有59例患者出现77例次术后并发症，手术死亡2例。Logistic回归分析，标准POSSUM的18项指标中17项及肺功能、肿瘤分期对术后并发症的发生有统计学意义（*P* < 0.05），年龄对术后死亡有统计学意义（*P* < 0.05）。在标准POSSUM评分中，并发症组与无并发症组的评分比较，差异有统计学意义（*P* < 0.01）。在改良POSSUM评分中，并发症组与无并发症组的评分比较，差异有统计学意义（*P* < 0.01）。改良POSSUM较标准POSSUM对术后并发症发生有更好的预测价值，两组AUC比较，差异有统计学意义（*P* < 0.01）。但改良POSSUM对手术死亡的预测值过高。

**结论:**

改良POSSUM评分对老年NSCLC术后并发症发生有较好的预测价值，可为决策手术治疗提供依据。

## Abstract (en)

**Background and objective:**

For the assessment of elderly patients can tolerate lung cancer operation, there is no clear standard. To evaluate the clinical validity of POSSUM (Physiological and Operative Severity Score for the Umeration of Mortality and Morbidity) in elderly non-small cell lung cancer (NSCLC) surgery patients, we want to provide an important basis for operation treatment decisions.

**Methods:**

A total of 138 patients, with 88 males and 50 females, with elderly NSCLC surgery between December 2007 and December 2013, are included in PLA general hospital. Using the multivariate Logistic regression analysis, we evaluate the value of each factor on the actual postoperative complications mortality and morbidity. The scorings on standard POSSUM and modifed POSSUM in the complication group are compared with the non-complication group using the group *t* test. Drawing receiver operating characteristic (ROC) curve in standard POSSUM group and modifed POSSUM group, calculating the area under the curve (AUC), AUC in standard group is compared with modifed group using *t* test. Judge if the modifed POSSUM prediction is consistent with the actual mortality and morbidity.

**Results:**

Among 138 patients, there were 77 postoperative complications in 59 patients, 2 cases of death. According to the Logistic regression analysis, 17 of 18 factors in standard POSSUM, pulmonary function, different TNM stage are predictors for postoperative complications (*P* < 0.05). Age is a predictor for postoperative death (*P* < 0.05). In the standard POSSUM scoring, actual complication group compared with non-complication group, the difference is statistically signifcant (*P* < 0.01). In the modifed POSSUM scoring, complication group is compared with non-complication group, the difference is statistically signifcant (*P* < 0.01). Compared with the standard POSSUM, the modifed POSSUM has beter predictive value on postoperative morbidity, and the comparison of AUC between the two groups is statistically signifcant. But the later shows the overpre-dicted mortality (*P* < 0.01).

**Conclusion:**

The modifed POSSUM has a good predictive value on postoperative complications in elderly NSCLC surgery patients, so it can provide the basis for decision-making operation treatment.

据中国肿瘤登记中心2012年报显示：肺癌已位居全国恶性肿瘤发病的第一位^[1]^。随着社会老龄化，肺癌在老年人中也有较高的发病率，非小细胞肺癌(non-small cell lung cancer, NSCLC)的中位发病年龄为70岁^[2]^。老年人早期的NSCLC首选治疗方法为手术治疗^[3]^。而老年肺癌患者是否选择手术治疗，不仅要考虑到患者的实际年龄，更需要考虑患者的全身状况、内科合并症及生命预期、手术获益与风险等情况^[4]^。目前评价老年患者能否耐受肺癌手术的方法尚无明确标准。POSSUM(Physiological and Operative Severity Score for the Umeration of Mortality and Morbidity)评分系统是Copeland^[5]^于1991年提出的，最早用于预测胃肠道手术后并发症发生率和病死率。它包含12项生理学指标，分别是：年龄、心脏征象、呼吸系统、收缩压、脉率、Glasgow昏迷评分、血红蛋白、白细胞、尿素、钠、钾、心电图。及6项手术严重度指标，分别是：手术范围、30天内手术次数、失血量、腹腔感染、恶性肿瘤和手术类别。以上指标按其程度分为1分、2分、4分、8分。1分为最轻，8分为最重。而将POSSUM评分用于老年肺癌手术患者的评估，目前报道甚少。本研究拟通过标准POSSUM评分及改良的POSSUM评分对老年肺癌手术患者术后风险进行预测，并与实际手术并发症发生率和病死率进行比较，来衡量改良POSSUM评分在老年肺癌手术并发症和手术死亡中的预测价值。

## 1 资料与方法

### 1.1 一般资料

收集从2007年12月-2013年12月解放军总医院连续住院行肺癌手术的老年患者138例。其中男性88例，女性50例。年龄65岁-86岁，平均(71.2±11.4)岁。术后病理诊断：细支气管肺泡癌62例，腺癌36例，鳞癌29例，腺鳞癌3例，大细胞癌3例，类癌2例，未分化癌2例，复合性癌1例。TNM分期：Ⅰa期56例，Ⅰb期41例，Ⅱa期19例，Ⅱb期11例，Ⅲa期6例，Ⅲb期5例。

### 1.2 手术方式

肺叶切除术66例，其中胸腔镜辅助下肺叶切除术50例；肺楔形切除术38例，其中胸腔镜辅助下肺楔形切除术26例；肺段切除术34例，其中胸腔镜辅助下肺段切除术23例。

### 1.3 纳入与排除标准

纳入标准：年龄≥65岁；病理证实为NSCLC；术前未行放化疗治疗。排除标准：年龄 < 65岁；病理证实为小细胞肺癌；术前已行放化疗治疗；合并全身转移的Ⅳ期患者；肺转移癌。

### 1.4 研究方法

#### 1.4.1 资料的收集

根据标准POSSUM评分系统收集每位患者术前24 h的12项生理学指标及6项手术严重度指标。并收集文献报道中可能影响并发症发生和手术死亡的因素：肺功能指标包括：第1秒用力呼气率(forced expiratory in the frst second, FEV_1_%)和肺一氧化碳弥散率(diffusing capacity of the lung for carbon monoxide, DLCO%)、手术时间、手术方式、病理分型、肿瘤分期。并按照标准POSSUM和改良POSSUM分别进行评分。根据COPELAND方程^[5]^计算术后(30 d内)并发症发生概率(R_1_)和手术死亡概率(R_2_)的预测值。并统计术后实际并发症发生例数和手术死亡例数。COPELAND方程如下：

ln[R_1_/(1-R_1_)]=-5.91+(0.16×生理学评分)+(0.19×手术侵袭度评分)

ln[R_2_/(1-R_2_)]=-7.04+(0.13×生理学评分)+(0.16×手术侵袭度评分)

#### 1.4.2 病例分组

以实际有无手术并发症分为并发症组和无并发症组，因死亡例数少，死亡病例未单独分组。依照标准POSSUM及改良POSSUM评分计算出R_1_和R_2_的预测值，分为预测并发症组和实际并发症组；预测病死率组和实际病死率组。

### 1.5 统计学方法

采用SPSS 17.0和MedCalc 13.0.0统计软件进行统计分析。计量资料采用Mean±SD表示，预测并发症发生率和病死率用百分比表示。各影响因素对术后实际并发症发生率和病死率的影响，采用二值多元*Logistic*回归分析。采用成组*t*检验对有、无并发症组的标准POSSUM评分值进行比较。同法，比较改良POSSUM评分值。绘制标准POSSUM和改良POSSUM的受试者工作特征曲线(receiver operating characteristic curve, ROC)，计算曲线下面积(area under the curve, AUC)，两组间AUC比较采用*t*检验。计算改良POSSUM评分预测和实际并发症发生率和病死率的符合度。*P* < 0.05为差异有统计学意义。

## 2 结果

### 2.1 术后并发症与死亡情况

共有59例患者发生术后并发症77例次，发生率为42.8%。其中发热(排除术后吸收热)17例，术后出血2例，气胸8例，肺不张5例，肺部感染5例，脓胸1例，呼吸衰竭3例，哮喘1例，皮下气肿6例，大面积肺栓塞1例，急性心衰2例，高血压4例，心律失常6例，急性冠脉综合征5例，不全性肠梗阻2例，高胰酶血症2例，急性胆囊炎1例，痛风1例，电解质紊乱2例，谵妄2例，急性肾功能衰竭1例。术后死亡2例，其中因急性大面积肺动脉栓塞死亡1例；因术后出血及肺部感染导致呼吸衰竭死亡1例。

### 2.2 *Logistic*回归分析

以术后实际并发症发生率及病死率为因变量，标准POSSU M的12项生理学指标和6项手术严重度指标及FEV_1_%、DLCO%、手术时间、手术方式、病理分型、肿瘤分期为自变量进行*Logistic*回归分析。标准POSSUM的18项指标除glasgow昏迷评分外，余下17项指标和FEV_1_%、DLCO%及肿瘤分期对术后并发症发生有统计学意义(*P* < 0.05)。而手术时间、手术方式、病理分型对术后并发症发生率无统计学意义(*P* > 0.05)。仅年龄对术后病死率有统计学意义(*P* < 0.05)。

结合以上统计分析结果，在原POSSUM评分基础上，将FEV_1_%和DLCO%赋值带入生理学指标，胸腔感染赋值带入手术侵袭度指标，建立改良POSSUM评分系统(表 1)。手术时间、手术方式因无统计学意义未赋值带入。肿瘤分期按标准POSSUM赋值带入。

**1:** 肺功能及胸腔感染赋值 The assignment of lung fuction and chest infection

	1 score	2 score	4 score	8 score
FEV_1_%	> 71	71-60	60-41	≤40
DLCO%	> 81	81-77	77-72	≤72
Infection	Non	Clear	Pus	Contents of gastrointestinal tract
FEV_1_: forced expiratory in the first second; DLCO: diffusing capacity of the lung for carbon monoxide.				

### 2.3 标准POSSUM和改良POSSUM生理学评分和手术严重度评分对术后实际并发症发生的影响

在标准POSSUM评分中，实际并发症组与无并发症组的生理学评分、手术严重度评分比较，差异有统计学意义(*P* < 0.01)。在改良POSSUM评分中，并发症组与无并发症组的生理学评分、手术严重度评分比较，差异有统计学意义(*P* < 0.01)(表 2)。

**2:** 有无并发症组的标准POSSUM和改良POSSUM评分比较 The comparison of standard POSSUM scoring and modified POSSUM scoring between with and without complication groups

	Complication (*n*=64)			Non-complication (*n*=74)	
	Physiological	Operative severity		Physiological	Operative severity
Standard	22.1±5.5^*^	11.6±0.7^#^		18.8±4.4	10.5±0.8
Modified	24.5±4.8^*^	12.1±0.9^#^		20.3±5.1	11.6±0.7
^*^vs physiological score of non-complication group, *P* < 0.01; ^#^*vs* operative severity score of non-complication group, *P* < 0.01. POSSUM: Physiological and Operative Severity Score for the Umeration of Mortality and Morbidity.					

### 2.4 POSSUM和改良POSSUM预测并发症发生率AUC的比较

标准POSSUM和改良POSSUM预测的术后并发症发生率与实际并发症发生分别进行比较并绘制ROC曲线，计算AUC，两组间AUC比较差异有统计学意义(*P* < 0.01)(表 3)。改良POSSUM的AUC更接近1，较标准POSSUM相比，其对术后并发症发生的预测价值更好。因实际死亡例数较少，无法进行预测病死率与实际死亡人数的ROC曲线绘制。

**3:** 标准POSSUM与改良POSSUM曲线下面积的比较 The comparison of AUC between standard POSSUM and modified POSSUM

	AUC	95%CI	*P* value
Standard	0.64±0.11	0.44-0.80	﹤0.01
Modified	0.73±0.09	0.54-0.87	
AUC: area under the curve.			

### 2.5 POSSUM评分预测并发症发生率和病死率与实际值的比较

POSSUM评分预测并发症发生率与实际并发症发生的O/E值波动在0.83-1.33。但改良POSSUM评分预测病死率与手术死亡的O/E值仅为0-0.50，存在过度预测(表 4，表 5)。

**4:** 改良POSSUM预测与实际术后并发症发生的例数对比 The comparison between predicted and actual complition cases

R_1_ (%)	*n*	Average R_1_ (%)	Predicted	Actual	O/E
0-10	2	6.2±1.84	0	0	1.00
11-20	18	15.5±2.55	3	4	1.33
21-30	44	27.8±2.87	13	17	1.30
31-40	10	35.0±5.65	4	4	1.00
41-50	17	44.1±2.31	8	7	0.88
51-60	32	55.8±3.34	18	17	0.95
61-70	9	64.2±2.82	6	5	0.83
71-80	4	72.3±3.51	3	3	1.00
> 80	2	85.1±4.16	2	2	1.00
R_1_: postoperative complications' incidence rate; O/E: observed to expected ratio.					

**5:** 改良POSSUM预测与实际手术死亡的例数对比 The comparison between predicted and actual death cases

R_2_ (%)	*n*	Average R_2_ (%)	Predicted	Actual	O/E
0-10	119	3.4±2.33	4	1	0.25
11-20	11	14.1±1.78	1	0	0
21-30	8	23.6±3.21	2	1	0.50
R_2_: mortality rate.					

## 3 讨论

标准POSSUM评分系统最早提出于1991年^[5]^。最新的*meta*分析显示对于肝胆胰手术来说，标准POSSUM评分过高预测了手术并发症和手术死亡^[6]^。而针对血管外科的V-POSSUM评分^[7]^；针对胃食道手术的O-POSSUM评分^[8]^；针对结直肠手术的Cr-POSSUM评分^[9]^却能更好地预测术后并发症发生及手术死亡。究其原因，手术技术的不断改进，特别是微创技术的广泛应用，使得术后并发症和手术死亡不断下降，故而较早提出的标准POSSUM预测值偏高。曾有报道由于微创技术的应用，平均年龄72岁的老年患者胸腔镜辅助下肺切除术的手术并发症发生率15%，手术死亡率由25年前的7%下降至0.8%^[10]^。其次，在标准POSSUM评分中一些与专科手术相关的参数并未纳入，以肝胆胰手术为例血清胆红素水平并未纳入在标准POSSUM评分中，也使得其预测率不准确^[11]^。在本研究中，我们根据*Logistic*回归分析结果，将肺通气指标FEV_1_%和肺弥散功能指标DLCO%及胸腔感染指标纳入改良POSSUM评分中，与标准POSSUM评分相比，其在术后并发症的预测上有更好的价值，两者AUC比较差异有统计学意义(*P* < 0.05)。但也应看到即使是改良POSSUM对手术死亡仍存在过度预测。

尽管手术治疗是老年早期NSCLC的首选治疗方法^[12]^，但是年龄仍是老年肺癌手术死亡的独立危险因素，一项3, 000例老年肺癌死亡率的调查显示^[13]^与年青人相比，30 d、60 d、90 d手术死亡率分别为3.6% *vs* 2.2%；4.1% *vs* 2.4%；4.7% *vs* 2.5%。但是结果不尽相同，Rivera^[11]^报道年龄与住院时间及术后并发症发生率无关。在本研究中，通过*Logistic*回归分析，年龄与术后并发症发生和手术死亡均相关。并且肿瘤TNM分期也与术后并发症发生率相关，这可能因为不同的TNM分期患者手术时间、手术方式、手术范围包括肺门纵隔淋巴结清扫的程度不同，直接影响到术后并发症的发生。值得提出的是目前NSCLC Ⅳ期的老年患者并不建议手术治疗，故本研究中并未纳入合并全身转移的老年NSCLC患者。

在我们的研究中，手术并发症和手术死亡与手术时间、手术方式及肿瘤的病理分型无关，这与国内的报道并不一致，杨等^[14]^报道手术时间是肺癌术后并发症发生和手术死亡的危险因素。分析原因本研究中多数患者采用的是胸腔镜辅助下的肺切除术，手术时间大多在2 h-3 h，大大降低了因手术时间过长，单侧肺通气时间长而造成的患侧肺萎陷、健侧肺过度通气带来的通气/血流比例失调。而马等^[15]^报道手术方式与肺癌术后并发症发生相关，也与本研究不一致。本研究中不同的手术方式，对术后并发症及手术死亡并无影响。由于国外文献^[16, 17]^报道全肺切除术可以使老年患者围术期并发症发生率及病死率增高，应尽量避免。故本研究在手术方式的选择上无全肺切除术。虽有回顾性报道^[18-20]^肺局限性切除术与肺叶切除术相比，术后生存期相当，但会有更好的肺功能。但本研究的观察时间仅局限在术后30 d内，由于观察时间较短不同手术方式所致的肺功能差异可能并未显现。

总之，改良POSSUM评分对老年NSCLC术后并发症发生有较好的预测价值，可为决策手术治疗提供依据。
